# No evidence of impaired gastric emptying in early Huntington‘s Disease

**DOI:** 10.1371/currents.RRN1284

**Published:** 2011-10-25

**Authors:** Carsten Saft, Jürgen Andrich, Marc Fälker, Sarah Gauda, Sina Küchler, Dirk Woitalla, Oliver Goetze

**Affiliations:** ^*^Department of Neurology, Huntington-Center NRW, Ruhr-University, St. Josef-Hospital, Bochum, Germany; ^‡^Department of Medicine 1, St. Josef-Hospital, University of Bochum, Germany; ^§^Department of Neurology, Huntington-Center NRW, Ruhr-University, St. Josef Hospital, Bochum, Germany; ^#^Department of Neurology, Huntington Center- NRW, Ruhr-University, St. Joseph Hospital, Bochum, Germany and ^**^Division of Gastroenterology and Hepatology; University Hospital Zurich; Zurich, Switzerland

## Abstract

Background: Several factors, such as dysphagia, an increased motor activity, increased metabolic rate and a hypermetabolic state have been discussed as contributing to weight loss even at the early stages of Huntington’s Disease (HD). Aim of this pilot study was to investigate gastric emptying as a possible reason for weight loss in HD.

Methods: 11 HD participants at early stages of the disease and matched controls were investigated by using the well-established and non-invasive 13C-octanoate breath test. The “Gastroparesis Cardinal Symptom Index” and the “Short-Form Leeds Dyspepsia Questionnaire” were used for clinical evaluation of gastroparesis or dyspepsia.

Results: When compared to standard values ​​given in literature and controls all HD patients had normal breath test results. There was no evidence of gastroparesis or dyspepsia. There was a correlation of breath test results with the cognitive and functional performance of HD participants.

Conclusion: According to our data, there is no evidence of impaired gastric emptying in early HD. We can not exclude that gastric emptying contributes to weight loss at more advanced stages of the disease.

Corresponding author: PD Dr. med. Carsten Saft, Department of Neurology, Huntington-Center NRW, St. Josef Hospital, Gudrunstrasse 56, 44791 Bochum, Germany, E-mail: carsten. Saft@ruhr-uni-bochum.de    

§ Carsten Saft and Jürgen Andrich contributed equally to this work 

## **Introduction:**

Weight loss is a main feature in Huntington’s disease (HD) and was found to be manifest even at early stages of the disease.[1] [2] [3] [4] [5] Multifactorial causes, such as decreased caloric intake due to dysphagia and a higher energy expenditure due to increased motor activity have been discussed as being a possible reason for weight loss especially at the advanced stages of the disease.[6] [7] [8] [9] [ [10] Using a whole body indirect calorimetry in both early stage HD patients and the R6/2 transgenic mouse model of HD, Goodman and colleagues were able to demonstrate that patients with early HD tended to have a negative energy balance for reasons not related to their movement disorder, which was paralleled in the transgenic R6/2 mice.[4] This leads to the assumption of an increased metabolic rate as a main reason for weight loss in HD, which is supported by other experiments in the transgenic R6/2 mice.[4] [11] [12] In a study investigating the direct relation between the number of CAG repeats in the mutant huntingtin gene and weight loss, Aziz and colleagues found a correlation between both of these factors and discussed a hypermetabolic state as being a reason for weight loss, occurring even at early stages of the disease.[13] They discussed a hypermetabolic state as being likely to stem directly from interference of the mutant protein with cellular energy homeostasis and thus reflecting fundamental pathologic mechanisms underlying HD and not to be secondary to hyperactivity. Since mutant Huntingtin (mtHtt) is not only expressed in the brain of HD patients, but also in the gastrointestinal (GI) tract, a recently published study investigated the GI tract in the R6/2 mice model for HD. This study describes a loss of enteric neuropepitdes, a decreased mucosal thickness and villius length and also an impaired gut motility, diarrhea, and malabsorption of food, suggesting that GI dysfunction plays an important role in weight loss in HD mice.[14]

In addition, gastrointestinal dysfunction is discussed as being the main reason for weight loss in Parkinson’s disease (PD).[15] In a study using a solid meal and the ^13^C-sodium octanoate breath test for measurement of gastric emptying in patients with PD, Goetze and colleagues found 88% of PD patients suufered from delayed gastric emptying when compared with controls. The severity of motor impairment was associated with gastroparesis.[16] Several other studies confirm an impaired gastric emptying in PD, some of them with a rate of 100% of PD patients.[16] [17] [18] [19] [20] [21] One study describes a 60% delay in gastric half emptying time in the PD patient group after a solid test meal using the non-invasive ^13^C-sodium octanoate breath test for evaluation of gastric emptying.[17] Neuropathological findings suggest enteric dysfunction to be one of the initial pathophysiological events in PD.[16] [22] Central and enteric nervous system involvement in PD is discussed as being a pathophysiologic basis for this dysfunction.[15]

Autonomic nervous dysfunction was found to be present in HD, too.[23] Thus, the aim of the current study was to investigate gastric emptying in early HD patients without medication as a possible additional reason for weight loss by using the well-established ^ 13^C-octanoate breath test.[16] [17] [18] [19] [20] [21] [24]     

## **Methods:**

### **Participants:**

11 manifest HD patients with genetically confirmed diagnosis and without any medication in at clinically early stages of the disease (Shoulson stage I/II) and 11 controls were recruited from the HD centre Bochum, Germany.[25] Participants with known concurrent gastrointestinal diseases or previous operations of the gastro-intestinal tract were excluded, as well as patients with other severe diseases, diabetes mellitus, severe respiratory dysfunction, and malignancies. Also participants with concurrent liver diseases or excessive alcohol consumption (50 g/d of ethanol) were excluded. All participants had lab parameters for ALT, AST, LDH, cholesterol and triglycerides within the normal range, as well as normal findings for the ultrasonography of the upper abdomen. Pregnant and breast-feeding women were excluded. All HD participants underwent neurological investigation and were scored according to the UHDRS items “motor scale” (MS), “total functional capacity” (TFC) “independence scale” (IS) and the items verbal fluency test, symbol digit test, interference test, color naming and color reading which were summarized as “cognitive score” (CS).[26] Fine motor skills were additionally measured by simple (tapping; higher motor impairment leads to lower test results) and complex (pegboard; higher motor impairment leads to higher test results) instrumental movement tests.[27] [28] [29] [30] The severity of depressive symptoms was assessed by using the Beck’s depression inventory (BDI) and Hamilton depression rating scale.[31] [32] Clinical characteristics of all HD patients are given in table 1. In addition we calculated the disease burden score (DBS = [CAG repeat - 35.5] x age) for each subject.[33] The study was approved by the ethic committee of the Ruhr-University Bochum, Germany (registration-number 2719). Participants gave informed written consent according to GCP/ICH.  

**Table:** 

**Parameter**	**HD Participants**	**Controls**
Age [yr]	42.4 ± 8.4 (29-57)	48.9 ± 9.6 (38-69)
Gender (male/female)	3/8	3/8
BMI	22.5 ± 3.5 (16-30)	26.5 ± 6.4 (19-42)
Weight [kg]	63.6 ± 14.1 (42-85)	84.5 ± 22.5 (54-128)
Height [cm]	166.8 ± 10.1 (153-183)	178.2 ± 9.4 (164-190)
AO motor	39 ± 8.6 (25-51)	-
AO psychiatric	38 ± 20.9 (29-50)a	-
CAG expanded	45 ± 2.9 (42-51)	-
Disease burden score	386.59 ± 66.06 (273-483)	-
Disease duration [yr]	4.2 ± 2.5 (0.1-9)	-
		
UHDRS MS	30.8 ± 18.7 (5-72)	-
UHDRS TFC	10.2 ± 1.9 (7-12)	-
UHDRS IS	81.8 ± 9.8 (70-100)	-
UHDRS CS	195.1 ± 79.0 (98-346)	-
Verbal fluency	22.5 ± 18.2 (4-69)	-
SDMT	27.2 ± 10.6 (16-44)	-
Stroop color	47.1 ± 17.1 (26-74)	-
Stroop word	68.1 ± 22.8 (32-100)	-
Stroop interference	29.5 ± 16.1 (10-59)	-
Hamilton	12.8 ± 10.0 (1-26)	-
Beck depression inventory	12.3 ± 13.4 (0-39)	-
		
Tapping dominant	129.2 ± 44.8 (47-198)	-
Tapping non dominant	99.4 ± 33 (38-161)	-
Pegboard dominant [sec]	68.6 ± 24.8 (42.2-120.9)	-
Pegboard non dominant [sec]	80.9 ± 40.2 (43.9-184.0)	-

**Table 1:** Clinical characteristics of 11 HD patients and 11 matched controls; values are given as mean ± SD; range (min-max) in brackets; Abbreviations: BMI – body mass index, yr – years, AO – age at onset, ^a^ n = 6; UHDRS – unified Huntington´s disease rating scale, MS – motor score TFC – total functional capacity, IS – independence scale, CS – cognitive sum score, SDMT – symbol digit modalities test; sec – seconds. * – significant differences.   

### **Test meal and** ^**13**^ **C-octanoate breath test technique:**

The ^13^C-octanoate breath test was used in the same way as described earlier.[16] [17] [18] [34] In summary: After an overnight fasting each participant received a solid test meal consisting of an egg omelet of one egg, 60 g of white bread, 5 g of margarine and 150 ml of water (14 g of proteins, 26 g of carbohydrates and 9 g of fat, 241 kcal) labeled with 100 mg of 13C-sodiumoctanoate (chemical purity of 99,7 % and an isotopic purity of 99,1 %) at 8 AM. Breath samples, which were expired in close aluminized plastic breath bags of 50 ml content were obtained before substrate administration at baseline and after 10, 20, 30, 45, 60, 75, 90, 105, 120, 135, 150, 165, 180, 200, 220 and 240 minutes. The subjects were kept in a relaxed sitting position during the octanoate breath test (OBT). Physical activity was restricted during the test. All subjects consumed their test meal within 10 minutes. The ^13^C/^12^C isotope ratio of the breath samples was analysed by isotope-selective nondispersive infrared spectrometer (NDIRS). The results were both expressed as delta (δ) value per mil (‰) and delta over baseline (dob = δ_s_ - δ_0_). Definition of the δ-value: δ_s_ = (R_S_/R_PDB_ -1) x 1000 [‰] with R_s_ = ^13^C/^12^C isotope ratio in CO_2_ in breath and R_PDB_ = 0.0112372 = isotope ratio in reference (PDB = PeeDeeBelmnite, South Carolina; δ_0_ = isotope ratio at baseline). 

### **Mathematical analysis of** ^**13**^ **CO**_**2**_ **excretion curves and statistical analysis:**

As regards the measuring of the proportion of the ^13^C-sodium octanoate given by mouth that is metabolised the results were expressed as a percentage dose of ^13^C recovered (PDR) over time for each time interval from which the cumulative PDR (cPDR), obtained by numerical integration from PDR values, was calculated for each time interval. This calculation is based on the formula as proposed by Ravussin.[35] CO_2_ production rate was assumed as being 300 mmol per unit of body surface area per hour. The body surface area was calculated using the Haycock weight-height formula.[36] The evaluation of the OBT for gastric emptying was done by non-linear regression analysis of the ^13^CO_2_-excretion curves (PDR) with the formula PDR(t) = at^b^e^-ct^. The expression ln a, as gastric emptying coefficient (GEC) is a reliable parameter to describe the rate at which the stomach empties. The percentage of ^13^CO_2_ cumulative values was fit using a model given by the formula cPDR(t) = m(1-e^-kt^)^ß^, where y is cPDR at time t in hours and m, k and ß are regression estimated constants, with m being the total amount of ^13^CO_2_ when time is infinite. Half gastric emptying time (t_50_) was calculated by taking PDR(t) equal to m/2 in the PDR equation which is expressed as t_50_ = (-1/k)ln (1-2^-1/ß^). The Lag phase is expressed as t_lag_= 1/klnß.[37] Statistical analysis was carried out as a descriptive evaluation of GEC, t_50_ (min), tl_ag_ (min) and t_peak_ (min) and characteristics of participants (mean ± SD). 

### **Gastroparesis Cardinal Symptom Index (GCSI) and Short-Form Leeds Dyspepsia Questionnaire (SF-LDQ).**

The well-validated Gastroparesis Cardinal Symptom Index (GCSI) was used for clinical evaluation of gastroparesis symptoms. GCSI quantifies nine symptoms in the three different subscales: nausea and vomiting, postprandial fullness, and bloating.[38] In addition patients were asked about the frequency and severity of their stomach complaints, heartburn, burping and nausea symptoms according to the Short-Form Leeds Dyspepsia Questionnaire (SF-LDQ).[39]

The data analysis and statistics were performed by using the commercial software program SPSS statistics 19. All measured parameters and clinical data were first analysed descriptively and they were when presented as mean ± SD. Normality of distribution of the data was tested with the one-sample Kolmogorov-Smirnov test. Data were analyzed using the independent t-test for comparison between HD participants and controls. Pearson correlation analysis was used for exploratory statistical calculations of the normal distributed data.       

## **Results:**

As expected HD participants had a lower body mass index compared to controls. One HD patient had underweight with a body mass index of 16. There were however, no significant differences between groups concerning any of the clinical data (table 1). Breath test results and clinical data showed normal distribution except for gender.

Results of the ^13^C-sodium octanoate breath test are given in table 2. ^13^CO_2_-excretion curves (PDR) and the percentage of ^13^CO_2_ cumulative values (cPDR) showed normal excretion of ^13^C. For PDR only PDR_max_ for the maximum amount of ^13^CO_2_-excretion reached during testing time is listed in table 2. There were no significant differences compared to controls for the values decisive for the evaluation of gastric emptying, such as PDR _max_, cPDR, GEC, t_50_ (min) and tl_ag_ (min; see table 2). Compared to standard values ​​given in literature, the most important parameters t_50_ and tl_ag_ were within normal range (t_50_ < 200 min and tl_ag_ <130 min; no data is available in literature for GEC, cPDR and PDR_max_) and none of the patients had abnormal breath test results (see figure 1).[24]

**Table:** 

**OBT Parameter**	**Results HD**	**Results Controls**
PDR _ max_	9.76 ± 2.86 6.32 – 14.18	9.85 ± 2.61 5.58 – 14.09
cPDR	23.64 ± 7.92 (14.72 – 37.0)	25.38 ± 7.92 (15.51 – 34.94)
GEC	2.96 ± 0.84 (0.95 – 3.77)	2.89 ± 0.36 (2.42 – 3.27)
t_ 50_	129.26 ± 38.84 (77.15 – 197.60)	135.88 ± 22.27 (95.74 – 167.47)
t _ lag_	85.45 ± 25.14 (55.95 – 123.42)	80.74 ± 17.33 (56.31 – 109.05)

**Table 2** ^**:**13^C-sodium octanoate breath test results; values are given as mean ± SD; range (min-max) in brackets, Abbreviations: PDR_max_ for the maximum amount of ^13^CO_2_-excretion reached during testing time [%]; cPDR – cumulative exhaled ^13^CO_2_ (cPDR [%]) after 240 minutes; GEC – gastric emptying coefficient; t _peak_ – time to highest exhaled ^13^CO_2_ value [min]; t_ 50_ – half gastric emptying time [min]; t _lag_ – Lag phase [min]. * – significant differences.

**Figure:**
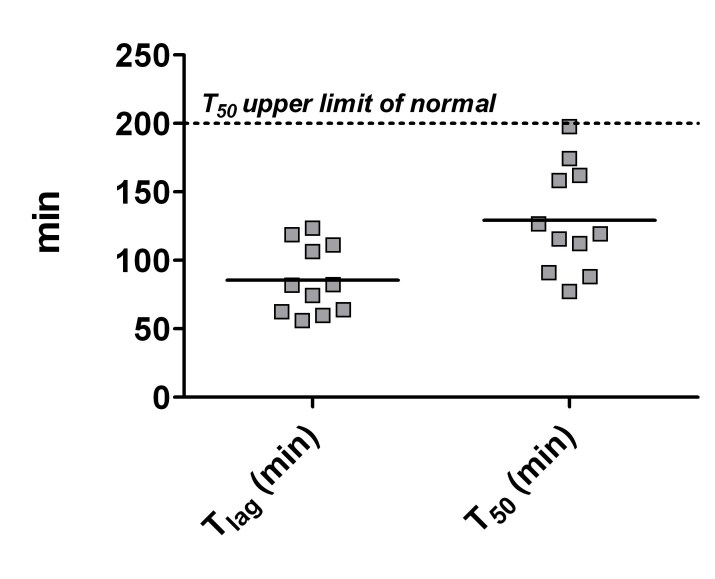

**Figure 1:  ** Gastric emptying of solids measured by ^13^C sodium octanoate breath test presented as individual lag phase (tl_ag_) and gastric half emptying time (t50) in 11 HD participants (controls not shown). The normal t_50_ range reported from literature (<200 min) is shown by the dotted line. A normal t_lag_ range is reported to be below 130 min.[24]

Gastroparesis Cardinal Symptom Index (GCSI) was 0.3855 (SEM ± 0.48; range 0 – 1.28) and Short-Form Leeds Dyspepsia Questionnaire (SF-LDQ) was 0.8182 (SEM ± 1.83; range 0 - 6) for HD participants. Thus, both questionnaire results were in line with published data from healthy controls, without clinical evidence of gastroparesis or dyspepsia.[38] [39] GCSI was 0.3027 (SEM ± 0.30; range 0 – 0.83) and SF-LDQ was 2.273 (SEM ± 2.195; range 0 - 7) for controls. Differences were not significant (data not shown).

Explorative correlation analysis of breath test results given in table 2 with clinical symptoms  from table 1 showed no significant correlation, except for the cognitive sum score and t_50_ (p 0.018, r -.692) and tl_ag_ (p 0.019, r -.688), as well as for PDR_max_ and the total functional capacity (TFC; p 0.014, r .712; no analysis of the cognitive subtests was done; see figure 2). Especially no correlation to motor symptoms was found.

**Figure:**
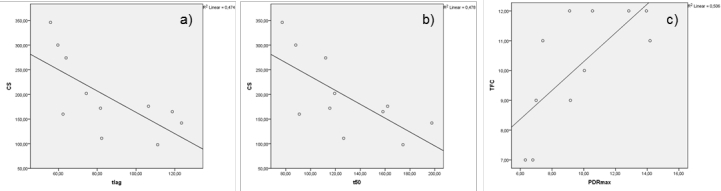

**Figure 2:  ** A strong relation can be seen between gastric emptying of solids measured by ^13^C sodium octanoate breath test, presented as individual  t_lag_ (a) and t_50_ (b) in minutes and the cognitive sum score of the UHDRS (t_50_ - p 0.018; r - .692 and t_lag_ - p 0.019; r - .688), as well as for PDR_max_ (c) and total functional capacity (TFC; p 0.014, r .712).  

The correlation analysis of GCSI and SF-LDQ with clinical symptoms showed no significant correlation with any of the clinical characteristics from table 1.   

## **Discussion:**

Several factors such as dysphagia, an increased motor activity, an increased metabolic rate and hypermetabolic state have been discussed as contributing to weight loss even at early stages of HD. In addition, a recently published study also suggested gastrointestinal tract dysfunction as a reason for weight loss in a Huntington mouse model, similar to findings for Parkinson’s disease (PD).[14] [15] [21] Several studies describe a delay in gastric emptying for 88% or even for up to 100% of PD patients.[16] [17] [18] [19] [20] [21] Contrary to this, in our pilot study on HD patients did not provide any evidence of impaired gastric emptying by using a solid meal and the ^13^C-sodium octanoate breath test. There were no significant differences compared to controls and also compared to standard values ​​given in literature all parameters were within normal range. In addition, we had no clinical evidence of gastroparesis or dyspepsia symptoms by using the “Gastroparesis Cardinal Symptom Index” and “Short-Form Leeds Dyspepsia Questionnaire” in our cohort. Thus, our data contrast with data for PD, but also with data for HD mice.

As a possible explanation, gastric emptying may only contribute to weight loss more severe stages of HD. R6/2 HD mice usually show a very rapid course of the disease. A recent published study investigating the GI tract in a R6/2 mice model carrying a mean of 204 CAG-repeats describes several GI abnormalities, including an increased water content in R6/2 compared to feces in wild type mice from 8 weeks of age. The fecal output as a percentage of food intake however, was only significantly increased at 12 weeks, but not at 8 weeks.[14] This indicates that the occurrence of malabsorption of nutrients plays an important role in weight loss in HD mice only in  the end stage. The study did not investigate early stages of the disease prior 8 weeks in the mice model.

An earlier study from our group describes a high prevalence of gastritis or esophagitis as an accidental finding during PEG-placement, as a possible indication of gastrointestinal tract dysfunction in HD patients at advanced stages of the disease.[40] The findings in this study were also correlated with the duration and severity of the disease, also suggesting that gastrointestinal tract dysfunction might occur later in the course of the disease. We presumed that influences from the disease itself as well as secondary mechanisms like medication and general disability may contribute.[40] It was also the case in this study that the focus was not on early symptomatic patients.

To summarize, the pilot data from our study suggest that impaired gastric emptying is not an early event in HD when compared to PD. We can not exclude that gastric emptying contributes to weight loss at more advanced stages of the disease.   

Surprisingly, we found a significant correlation for the cognitive sum score und the total functional capacity of the UHDRS and breath test results, such as t_50_ (47.8% of variance), tl_ag_ (47.4% of variance) and PDR_max_ which usually shows the most precise quantification (50.6% of variance; see figure 2). This was not expected, since OBT results were within normal range. Cognitive decline, however, is a very early event in the course of HD.[41] In fact the cognitive sum score from our HD participants showed a broad range from 98-346 points with a mean of 195.1 points indicating a cognitive impairment in most of the patients. It is well known that the performance in UHDRS cognitive tests declined during disease progression, as did the functional capacity (TFC), which is highly dependent on cognitive tasks.[42] [43] A decrement in mitochondrial function is discussed as contributing to age-dependent functional deficits in neurons and myocytes in normal aging and other neurological disorders, such as Alzheimer’s disease, accompanied with a cognitive decline.[44] [45] [46] Mitochondrial dysfunction is well known in HD and seems to be a relevant and early feature in the pathology.[47] [48] [49] Mutant htt (mtHtt) tends to aggregate in cytoplasm and nucleus of neurons as well as non-neuronal tissues including the liver.[50] [51] [52] [53] [54] [55] Within the mitochondria, octanoic acid undergoes b-oxidation. Octanic acid generates acetyl coenzyme A which enters the Krebs cycle and is oxidized to CO_2_. Therefore breath tests based on octanoate, usually used to assess gastric emptying, should also reflect mitochondrial function.[56] Thus,  one can speculate that a correlation of OBT results with results of cognitive tasks might reflect a parallel decline in cognitive and mitochondrial function. 

To our knowledge this is the first study dealing with gastrointestinal track dysfunction in HD in vivo. A limitation of our study is the relative small number of participants. To exclude drug effects we only included patients without any medication and without serious comorbidities. On the other hand, due to the fact that this is a very rare group of patients it is a strength of our study that we can exclude medication effects.  

## **Competing interests:**

The author(s) declare that they have no competing interests.

## **Ethics**

The local ethics committee of the university approved this study.

## **Acknowledgement**

We are grateful to all patients for participation. 

## **Funding information**

The study was supported by a FoRUM grant, University of Bochum (AZ: F506-2006). Oliver Götze was supported by the DFG (Gö 13582/1).
